# Measuring outcomes of rehabilitation among the elderly—a feasibility study

**DOI:** 10.3389/frhs.2023.1187713

**Published:** 2023-09-15

**Authors:** Laura J. Pitkänen, Jyri Niskanen, Antti Malmivaara, Paulus Torkki

**Affiliations:** ^1^ Department of Public Health, Faculty of Medicine, University of Helsinki, Helsinki, Finland; ^2^ Unit for Performance Assessment of the Health and Social Service System, Finnish Institute of Health and Welfare, Helsinki, Finland

**Keywords:** outcomes, effectiveness, real-effectiveness medicine, rehabilitation, elderly, Alzheimer’s, value-based healthcare

## Abstract

A feasible system for measuring patient outcomes of rehabilitation is required for assessing the real-world cost-effectiveness of rehabilitation. This study aims to assess the feasibility of measuring outcomes of rehabilitation among elderly individuals with early-stage Alzheimer's. We used the principles of Design Science to construct a set of metrics consisting of standardized PROM (Patient-Reported Outcome Measure) questionnaires, clinician-reported measures, and observational measures of functioning. We used standardized questionnaires whenever possible to ensure the validity and reliability of the questionnaires. The set of metrics was piloted on 16 individuals living at home with regular home care services. After the pilot, we further refined the set of metrics based on relevance, sensitivity to change, and applicability. We found that measurement was feasible and we propose the final set of metrics as a minimum set, which could be further improved upon by addition of metrics relevant to each subgroup of elderly individuals. We also found that using self-reported questionnaires in this population is not without difficulties. We therefore suggest that the role of informal caregivers be considered, and that accessibility of outcome questionnaires be improved.

## 1. Introduction

Rehabilitation of the elderly is often an underused intervention that has potential to improve functioning, prevent falls, and delay the need for more intensive services (1–4). From the point of view of the payer, cost-effectiveness of rehabilitation is key: whether rehabilitation can delay the need for hospice care long enough to offset the cost of rehabilitation. Calculating the costs is a relatively simple, while by no means negligible, task. What is missing from the equation, though, are the outcomes.

Much interest has been invested in measuring the outcomes of health care services in recent years and decades. Current consensus is that the set of measures should include—whenever possible—patient-reported measures to complement clinician-reported (ClinROM) and observational measures (5). Furthermore, a standardization of measures used is necessary for comparison (6). As the patient is often not the payer, it is also important to include the point-of-view of the payer, meaning costs and outcomes relevant to the payer (5). Furthermore, outcome data could be used in patient and intervention selection.

Another much-discussed aspect in healthcare is value-based purchasing. In theory, tying a part of the remuneration to outcome targets could incentivize the service producer to aim for best possible outcomes for the patient. Although there are many ways to design a value-based purchasing model (7) and it is unclear how exactly to design a successful one (8), a balanced set of measures is a necessary step.

Rehabilitation is a difficult setting for measuring outcomes, though, because the individual goals for each patient can vastly differ. The universal goal, according to the United Nations, is to enable persons with disabilities to “reach and maintain their optimal physical, sensory, intellectual, psychiatric and/or social functional levels, thus providing them with the tools to change their lives towards a higher level of independence” (9). WHO, on the other hand, emphasizes the integration of the individual and their environment (10), which is also reflected in their International Classification on Functioning (ICF) and its practical tools, ICF Core Sets.

Whether emphasis is on the individual or their surroundings or the interaction between the two, the ultimate goal of rehabilitation is to maximize the independence and autonomy of the individual. What differs from one individual to the other are the intermediary goals: what must happen for independence to be maximized. One way to quantify the attainment of individual goals in a comparable way is Goal Attainment Scaling (GAS) (11).

The outcome of rehabilitation of the elderly is often measured through mobility, which is a good proxy for Activities of Daily Living, ADL (12). A systematic review by Vermeulen et al. (13) found that a low ADL was preceded by weight loss, decrease in grip strength, and poor balance, among other things. ADL itself is also an often-used measure of rehabilitation outcomes in this population (1, 3, 13), as is the Functional Independence Measure (FIM) (1, 12).

There are also numerous examples of studies using Patient-reported Outcome Measures (PROM), both generic and specific, to measure the effectiveness of rehabilitation (12, 14–18).

Much like PROMs, measures of patient experience (Patient-Reported Experience Measures, PREM) also range from the generic [such as Net Promoter Score (NPS), widely used across industries] to the specific (such as PREM-SBC for secondary breast cancer). The dimensions measured are often similar in the generic and specific measures, and usually include communication, professionalism, etc. No PREM questionnaires developed specifically for rehabilitation or physiotherapy have been translated into Finnish.

Overall, literature is ripe with outcome measures used in studying rehabilitation. However, what is necessary in a randomized trial may not be feasible in real life - that is, the measuring in a randomized trial is much more comprehensive than what the professionals have time for in real life.

As populations in developed countries are getting older, the prevalence of different forms of dementia are increasing (19). Over 46 million people worldwide live with dementia, and this has been estimated to increase to over 130 million by 2050 (19). The total estimated cost of dementia was 1 trillion $ in 2018, estimated to double by 2013 (19). Alzheimer's disease is by far the leading cause of dementia, causing at least 70% of dementia cases worldwide (20).

The cognitive decline in people with dementia necessitates that particular attention be paid to accessibility (21). It is therefore that we chose Alzheimer's patients as our target group: a measuring system that is feasible in this population will likely be feasible in the wider elderly population.

Our primary aim was to assess the feasibility of a system for measuring outcomes, with elderly, early-stage Alzheimer patients as our target group. Thus, we primarily aimed to identify the relevant and applicable outcome metrics among already standardized and validated instruments. We then assessed their sensitivity to change in order to trim down the set of metrics based on this information.

## 2. Materials and methods

This is a qualitative feasibility study, which utilizes Design Science to design a system of outcomes measurement. In the context of this study, we define feasibility as consisting of three critical aspects: relevance, sensitivity to change, and applicability. In terms of relevance, we focus on the point-of-view of the patient and the professional, while also incorporating the point-of-view of the payer.

Design Science is solution-oriented and directly tied to practice and deals with the understanding and improving of an artifact that is intended to solve a problem (22). The artifact constructed in this study is a system for measuring the outcomes of rehabilitation in our chosen segment. We chose Design Science as a method because of the nature of the problem at hand: we set out to construct a system, an artifact to fulfill a practical need, and Design Science is a research paradigm for doing precisely that.

**Figure 1:**
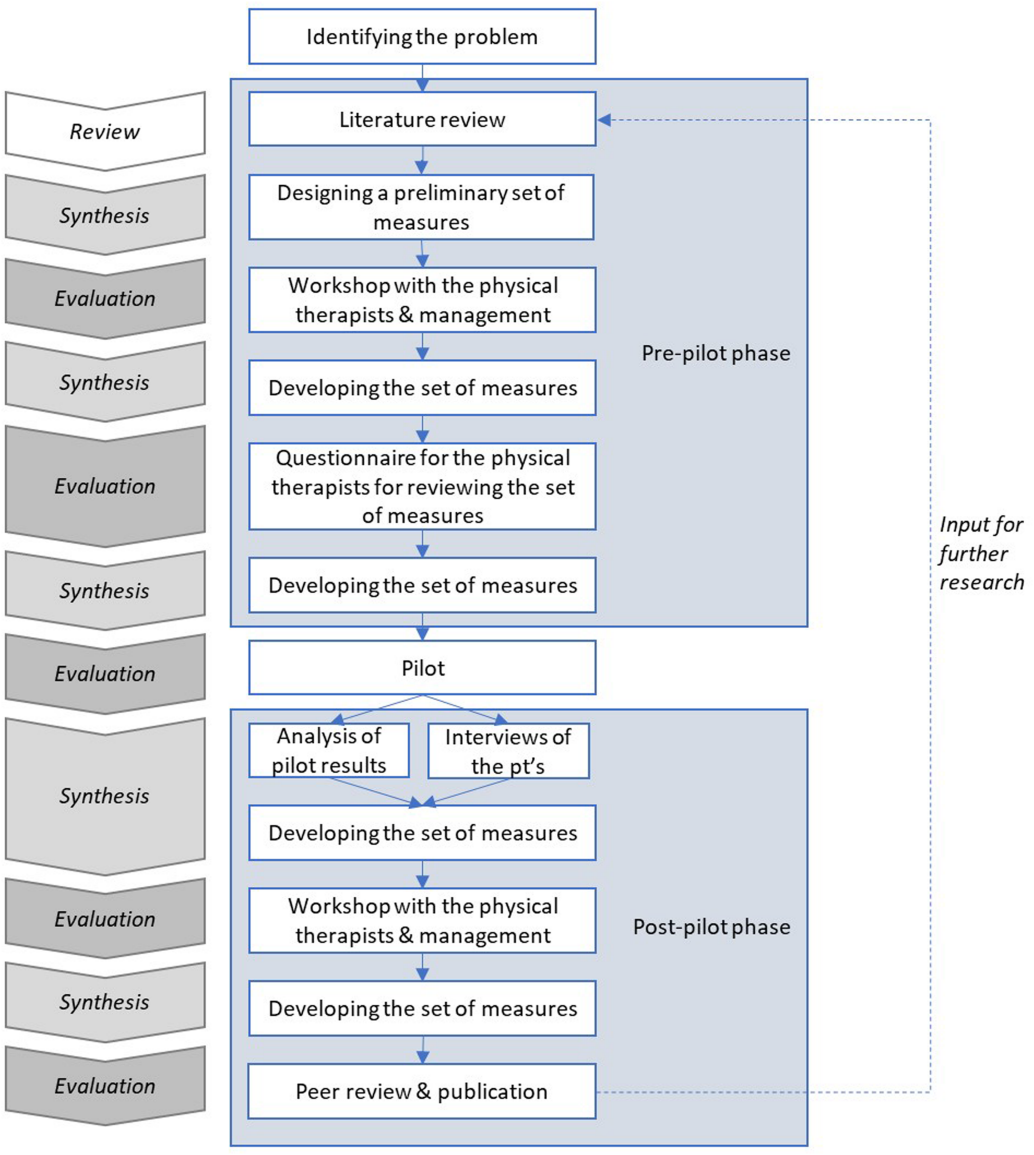
The steps of this study.

Taking a Design Science approach often involves a review of existing literature, followed by repeating cycles of synthesis and evaluation (23). Figure 1 describes the steps taken in this study, highlighting the cyclical nature of the process.

### 2.1. Pre-pilot phase

In developing the measurement model (the set of metrics and their measurement interval), we utilized the principles outlined in the literature above. We referred to the ICF Rehabilitation Core Set (24), which includes 30 items, and the TOIMIA Functioning Measures Database (25) which, among other things, bridges measures to ICF. Based on this information, we calculated the coverage of each alternative PROM, ClinROM, and observational measure, and of combinations thereof.

For PROM selection, we referred to Finnish TOIMIA guidelines for measurement of self-reported functioning (26) for both which questionnaires to use, and the time points of measurement. We also had workshops of the preliminary sets of metrics with the physiotherapists and with the management of the physical therapy provider.

Due to the heterogeneity of elderly people in terms of both health and activity, we decided to focus on a specific group of elderly. As elderly with Alzheimer's disease are prone to a decrease in their physical activity, possible interventions should yield positive outcomes. Thus, using measures on a rather homogenous group of patients we could consider if our set of metrics could be implemented on a wider scale with elderly patients.

The inclusion and exclusion criteria were designed to include elderly patients with a mild cognitive impairment and an adequate level of physical functioning which enables living at home with home care. Thus, the inclusion criteria were: being at least 65 years of age, retired from work, a diagnosis of Alzheimer's disease (ICD-10 codes G30.1, G30.9), living at home, and able to walk for 10 meters (independently or with a mobility aid). The exclusion criteria were other reasons for dementia: e.g., Parkinson's disease, Lewy body dementia, head trauma, and early-onset Alzheimer's (G30.0).

Using these criteria, we recruited 19 patients undergoing an individualized three-month rehabilitation intervention which consisted of cognitive and physical exercise with a physiotherapist (PT). One of the patients passed away during the study, and two were unable to complete the study for other reasons. Thus, our research population consisted of 16 individuals. The patients were 78–92 years of age (with a mean age of 85), and 69% (11) were female.

Patient recruitment and interventions commenced in early 2022. Interventions were conducted by two physiotherapists with 5 and 25 years of experience in rehabilitation of the elderly. Interventions completed in August 2022. Measurements were conducted both before and after the intervention.

### 2.2. Post-pilot phase

After interventions and data gathering had completed, we interviewed both of the physiotherapists separately (semi-structured interview and structured scoring of each metric). Each interview lasted about two hours and was conducted by two researhers, LP and JN. To increase the rigor of the qualitative part of the study, we asked the physiotherapists to score each questionnaire used. The assessment questions were formulated based on literature (27, 28) on patient experience of PROM usage—modified for professionals filling out the questionnaires in collaboration with the patient. The dimensions of metric assessment were relevance, understandability, usefulness, and overall usability. The questions used were as follows:

1. Were the questions relevant?
2. Were the questions understandable?
3. Were the questions useful (i.e., did they bring out topics that would not have been discussed otherwise?)?
4. Overall, would you use this questionnaire in the future?

The physiotherapists were asked to score each questionnaire on each dimension on a scale of 1 to 5.

We then had a workshop with the physiotherapists and with the management to finalize the set of metrics.

## 3. Results

### 3.1. Pre-pilot phase

We chose to adhere to standardized questionnaires to ensure the reliability and validity of the metrics.

In the TOIMIA guidelines (26), a measurement interval of three months was recommended, which coincided with the planned duration of the intervention. When choosing the measures used, we strived to minimize the amount of additional work for all stakeholders, especially the professionals. We therefore decided to include ADL, measured through the Barthel Index (29), as a ClinROM, since it is a part of RAI [Resident Assessment Instrument (30)] and therefore widely available. Similarly, for observational measures, we chose SPPB [Short Physical Performance Battery (31)] and Grip Strength, both already used by physiotherapists.

For a generic PROM, the TOIMIA guidelines (26) recommended either PROMIS Global Health (“PROMIS-10”), EuroHIS-8, or WHODAS 2.0 (12 items). We compared their ICF coverages: 23% for PROMIS-10, 37% for WHODAS 2.0, and 7% for EuroHIS-8. However, in combination with ADL/Barthel, SPPB and Grip Strength, coverage was 53% for either PROMIS-10 or WHODAS 2.0, and 40% for EuroHIS-8, meaning that there is considerable overlap in the coverage of different measures.

We also discussed questionnaire choice with the physiotherapists and agreed that while all three were somewhat negatively worded (for example, in WHODAS 2.0, all items are formulated as “In the past 30 days, how much difficulty did you have in: –”, thus emphasizing difficulties), PROMIS-10 was the least negative. Based on this, and the coverages mentioned above, we chose PROMIS-10, along with PASS (Patient Acceptable Symptom State) and GRC (Global Rate of Change), as recommended by TOIMIA (26).

For patient satisfaction measurement, we chose NPS (Net Promoter Score) for easy comparison, and additionally modified a short survey based on the patient satisfaction survey used by The Finnish Institute for Health and Welfare in their biennial national FinSote survey (32).

To measure obtained goals, GAS (GAS 1) (33) was chosen, as it is often used in Finland, including in rehabilitation.

Table 1 lists the metrics used in the pilot, and whether measurements were done both before and after the intervention, or only after.

**Table 1:** The pilot set of metrics.

Category	Metric	Before	After
Goals	GAS (goal attainment scaling)		X
Patient-reported measures	PROMIS Global Health (PROMIS-10)	X	X
	PASS (patient acceptable symptom state)	X	X
	*Itsearvioitu muisti, keskittymiskyky ja uuden oppimisen kyky* (a Finnish questionnaire on memory and cognition)	X	X
	GRC (global rate of change)		X
	Pain (on visual analogue scale)	X	X
	NPS (net promoter score)		X
	Customer satisfaction (a short survey of 8 items)		X
Clinician-reported measures	The barthel index/ADL (activities of daily living)	X	X
Observational measures	SPPB (short physical performance battery)	X	X
	Grip strength	X	X

### 3.2. Post-pilot phase

The idea was that the patients were to have a mild cognitive impairment only, so as to be able to fill out questionnaires independently. However, they turned out to be more cognitively impaired than expected. Thus, they were not able to fill out questionnaires by themselves, but required the help of the physiotherapist. Also, for the most part, the patients were unable to reflect on their symptoms for the last 7 days (or however many days the recall period of each questionnaire was), but rather tended to answer based on their experience on that particular day. Furthermore, many found the questionnaires too difficult and too complex in their wording and had trouble understanding the 5-point Likert scale used in many of the questionnaires. Some patients had a relative present, and the relative sometimes divulged information that conflicted the information divulged by the patient.

Based on the pilot data and the post-pilot interviews, Table 2 displays the fraction of patients with a change detected per metric (reflecting the sensitivity), and their average score for relevance and applicability (arithmetic average of the four scores given by the two physiotherapists; in total, eight scores per metric).

**Table 2:** Assessment of the suitability of the metrics.

Category	Metric	Sensitivity: % of patients with a change detected	Relevance and applicability: physiotherapist assessment (average)	Included in the final set of metrics	Comments/rationale for including
Goals	GAS (goal attainment scaling)	Not applicable	Not applicable	x	Gold standard in measuring goal attainment.
Patient-reported measures	PROMIS Global Health (PROMIS-10), physical health T-score	93%	3,75	x	Could be replaced with another generic PROM.
	PROMIS Global Health (PROMIS-10), mental health T-score	75%			
	PASS (patient acceptable symptom state)	19%	2,75		
	*Itsearvioitu muisti, keskittymiskyky ja uuden oppimisen kyky* (a Finnish questionnaire on memory and cognition)	60%	3,38		
	GRC (global rate of change)	69%	4,5	x	Potentially useful, even though difficulties arise in a cognitively impaired population.
	Pain (today, on a scale of 0–10)	44%	Not applicable		
Clinician-reported measures	The barthel index/ADL (activities of daily living)	44%	4,75	x	Included in RAI; thus, could be used as part of anamnesis/medical history in the future
Observational measures	SPPB (short physical performance battery)	75%	Not applicable	x	Widely used in measuring of physical functioning.
	Grip strength	94%	Not applicable	x	Widely used in measuring of physical functioning.

The final set of metrics was chosen based on the scores in Table 2, and interview data was utilized to form the rationale for inclusion. Based on the scores, we decided to leave out three metrics. PASS turned out to be too crude a metric (scale “yes” or “no”), especially for the cognitively impaired, whose answers are more likely to reflect their general attitude towards their functioning than any changes in it. The cognition-related PROM, while specifically aimed at cognitively impaired persons, seems unreliable—it might be useful if filled out by relatives of the patient. Pain is included in most generic PROMs and thus is redundant in this set of metrics—furthermore, cognitively impaired persons seem prone to answer using the extremes of the scale (9/12 gave either 0 or 10 pre-intervention, 10/15 post-intervention).

Table 2 shows which metrics were included in the final set of metrics, with comments on each metric.

## 4. Discussion

We aimed to assess the feasibility of a system for measuring patient outcomes in rehabilitation of early-stage Alzheimer's patients, and to design a set of metrics through an iterative process.

We were able to design a set of metrics which adheres to the critical aspects defined earlier: relevance, sensitivity for change, and applicability. The set of metrics is presented in Table 2. To the best of our knowledge, such a set of metrics for measurement of real-effectiveness of rehabilitation has not been published before. The ICHOM Older Person Standard Set (34) is somewhat similar, yet it does not focus specifically on the outcomes of rehabilitation and is thus too generic. The set we present is proposed as a minimum set, which could be further improved upon by the addition of some items discussed below. Furthermore, cost per patient is also needed to assess cost-effectiveness, which is essential for the payer.

We found that measurement was feasible. Despite the aforementioned problems, we were able to conclude a few things about which metrics seem to work for this population and which do not.

It would seem that patients with cognitive impairment, even if it is mild, need simpler questionnaires. This is congruent with the findings of Kramer & Schwartz (35), who suggest that the field of rehabilitation could benefit from considering the cognitive accessibility of PROM use. In practice, we suggest that both the questions and the multiple choice options need to be simpler. One way to achieve this could be by using a visual scale.

One barrier to adaptation of PROMs in routine use is patient burden. In the field of maternity care, a systematic review by Chen et al. (36) found that one factor hindering the acceptability of PROM collection was that the patients were concerned about the length of the questionnaires and the inadequacy of instructions. They offered suggestions for increasing response rate, such as explaining the purpose of the questionnaires to the patients and giving clear instructions, which could be applicable in the elderly population as well. In this study, physiotherapists collected PROMs through interviews, and thus we cannot comment on patient burden as such. We did strive to minimize patient burden by trimming down the set of metrics to a minimum, but real-life response rate remains to be seen.

People with Alzheimer's disease tend to have difficulties with abstract thinking and understanding of text, which are both essential for filling out questionnaires. Assistance and explanation with questions from different surveys were needed from a physiotherapist and/or a relative. Including observational measures in our set of metrics helps attain a more comprehensive view of changes in patients' well-being. This reduces reliance on one source of information and the impact of cognitive abilities of the patient. On the other hand, using a more heterogeneous group of elderly patients with a wider range of cognitive abilities would reduce the problem of written questionnaires. Nonetheless, using a questionnaire tailored for elderly patients with cognitive impairments should be further studied.

Also, we found that for many of the patients the most important outcome was an increase in mobility and specifically confidence in leaving one's home. Future studies could benefit from questionnaires measuring this phenomenon. CONFbal is a questionnaire which measures balance confidence (37), which could be one component of the phenomenon. However, we suggest that confidence in leaving one's home is about more than just balance, particularly for cognitively impaired individuals.

While some metric of personal goals and their attainment is needed, GAS is highly person-dependent and thus gives uneven results. Some service providers also consider it “too unwieldy for routine use” (38). On the other hand, some studies have found GAS to be more responsive to change than standardized outcome measures: Stole and Rockwood have studied this both in a geriatric setting (39) and in a cognitive rehabilitation setting (40). For lack of a better measure, we decided to include GAS in the final set of measures. However, we believe that standardization of goals and attainment levels, such as that developed by the Geriatric Assessment and Rehabilitation Unit some 25 years ago (38), could improve its uniformity in the future.

It should be noted that the changes mentioned in Table 2 can be small depending on the sensitivity of the metric. Therefore, the fraction of patients with change detected probably reflects two things: the sensitivity of the metric and the suitability of the metric for this particular population and context. For example, PROMIS-10 uses a scale of 0–100, while PASS uses a scale of 0–1, which may partly affect the sensitivity of the questionnaire. In future studies, we suggest that clinically meaningful changes should be assessed and taken into consideration. Furthermore, in a cognitively intact population the QRC question could be used to estimate the meaningfulness of change much like Osoba et al. (41) have used Subjective Significance Questionnaire to assess the subjective significance of changes in PROMs.

The physiotherapists involved in this study reported that there was sometimes a discrepancy between the views of the patient and their relatives regarding the health and well-being of the patient. This is probably attributable in part to the cognitive deterioration of the patients, but the physiotherapists also noted that sometimes the relatives’ judgement can be affected by their beliefs and attitudes: their view (often more negative than that of the patient) is not necessarily correct.

Relatives could also take a bigger role in helping patients fill out questionnaires. In this study the questionnaires were filled out with the help of the physiotherapist. In real life relatives could fill this role. However, as mentioned in the preceding paragraph the possible bias of the relative may affect results, which must be considered.

Overall, the pilot results presented many surprises. The usefulness of the metrics and the feasibility of measuring were affected by many unforeseen details. We feel that this emphasizes the importance of a feasibility study, particularly in populations of compromised functioning: without piloting the questionnaires it is impossible to know what will work.

Outcomes data can be used in estimating the real-effectiveness (42, 43) of a rehabilitation intervention when benchmarking between providers. However, our study did not aim to provide outcomes data to assess the effectiveness of the intervention.

The set of metrics we present includes assessment of functioning by both the patient and the professional. The scoping review by Ravn et al. (44) analyzes 26 trials, of which 10 measured either ADL of QoL, 8 measured both, and 8 measured neither. In the 2023 Cochrane review on cognitive rehabilitation for people with dementia (45) 5 out of 6 trials measured quality of life. It seems that while ADL and QoL are often-used outcome metrics in studies on this subject, there is still a lack of comprehensive measuring of outcomes from multiple points-of-view. A set of metrics comprising different points-of-view could improve the evidence base of rehabilitation of the elderly.

### 4.1. Limitations of the study

We aimed to measure MMSE (Mini Mental State Examination) in addition to the Finnish questionnaire on memory, so we could compare the patient-reported score with the MMSE, which is clinician-reported. However, due to human error (communication breakdown between researchers and the physical therapy provider), MMSE was not measured.

Furthermore, as only 7 out of 16 patients had finalized GAS forms, we decided to disregard these in the analysis. Due in part to the untimely passing of one of the physiotherapists during the study, the reasons for the incomplete filling out of GAS questionnaires remain unclear.

Notably, for some of the patients rehabilitation began in the winter months and ended in the spring, while for others rehabilitation began in the early summer and ended in late summer. In Finland, weather conditions vary greatly between the seasons: in the winter, streets can be icy and slippery, rendering many elderly people unable to leave their home on their own. Spring, on the other hand, is ideal for outdoor activity. Likewise, summer can be quite hot at times and as Finnish homes rarely have air conditioning many elderly people suffer from the heat. Thus, the timing of the rehabilitation intervention could affect the results.

The relatively advanced state of cognitive deterioration among the patients meant that they had difficulty recalling their symptom state for the last 7 days as required by some of the questionnaires used. Thus, the results may indicate the difference between the situation on a particular day rather than a true change in their symptom state within those three months. Therefore, random factors may have had a greater effect than they would among cognitively sound individuals.

As patients filled out the questionnaires with the help of their physiotherapist—who was also the provider of the service being evaluated—there may be some bias in the results, especially regarding customer satisfaction.

## 5. Conclusions

We found that measuring the outcomes was feasible. We were able to design a set of metrics which is relevant, sensitive for change, and applicable. The iterative process we employed provided results quite quickly. A similar process could also be useful in developing other such sets of metrics, such as metrics used in national quality registers.

Feasibility is ensured by using a concise set of metrics devoid of overlapping items. Using the ICF Core Set allowed us to maximize coverage while minimizing overlap.

A feasible system for measuring patient outcomes of rehabilitation is a step towards assessing the real-world cost-effectiveness of rehabilitation. This, in turn, could be used to optimize rehabilitation use in order to achieve better quality of life for elderly patients, and lower total cost for the payer. It could also be a step forward in outcomes-based payment systems.

Future studies could benefit from the inclusion of informal caregivers as a source of information regarding patients’ health and wellbeing. This can be achieved using standardized questionnaires and/or conducting interviews. Some quality-of-life measures offer different versions of the questionnaire for the informal caregivers.

## Data Availability

The datasets presented in this article are not readily available because Finnish law. Requests to access the datasets should be directed to laura.j.pitkanen@helsinki.fi.

## References

[1] Crocker T Forster A Young J Brown L Ozer S Smith J Physical rehabilitation for older people in long-term care. Cochrane Database Syst Rev. (2013) (2):CD004294. 10.1002/14651858.CD004294.pub3 23450551 PMC11930398

[2] Gillespie LD Robertson MC Gillespie WJ Sherrington C Gates S Clemson L Interventions for preventing falls in older people living in the community. Cochrane Database Syst Rev. (2012) 2012(1):CD012424. 10.1002/14651858.CD007146.pub3 PMC8095069 22972103

[3] Daniels R van Rossum E de Witte L Kempen GIJM van den Heuvel W. Interventions to prevent disability in frail community-dwelling elderly: a systematic review. BMC Health Serv Res. (2008) 8:278. 10.1186/1472-6963-8-278 19115992 PMC2630317

[4] Melin AL Håkansson S Bygren LO. The cost-effectiveness of rehabilitation in the home: a study of Swedish elderly. Am J Public Health. (1993) 83(3):313–474. 10.2105/AJPH.83.3.356 8438972 PMC1694665

[5] Larsson S. The patient priority. Solve health care’s value crisis by measuring and delivering outcomes that matter to patients. New York: McGraw Hill (2022) 56, p. 65–7

[6] Porter ME Larsson S Lee TH. Standardizing patient outcomes measurement. N Engl J Med. (2016) 374(6):504–6. 10.1056/NEJMp1511701 26863351

[7] Cattell D Eijkenaar F. Value-based provider payment initiatives combining global payments with explicit quality incentives: a systematic review. Med Care Res Rev. (2020) 77(6):511–37. 10.1177/1077558719856775 31216945 PMC7536531

[8] Damberg CL Sorbero ME Lovejoy SL Martsolf GR Raaen L Madel D. Measuring success in health care value-based purchasing programs. Rand Health Q. (2014) 4(3):9. 28083347 10.7249/rhq-04-03-09 PMC5161317

[9] UN. (1993). The standard rules on the equalization of opportunities for persons with disabilities. United Nations 48th session on 20 December 1993. New York.

[10] WHO. Disability prevention and rehabilitation. Report of the WHO expert committee on disability prevention and rehabilitation. Geneve: World Health Organization (1980).

[11] Turner-Stokes L. Goal attainment scaling (GAS) in rehabilitation: a practical guide. Clin Rehabil. (2009) 23:362–70. 10.1177/0269215508101742 19179355

[12] Kehusmaa S Autti-Rämö I Valaste M Hinkka K Rissanen P. Economic evaluation of a geriatric rehabilitation programme: a randomized controlled trial. J Rehabil Med. (2010) 42:949–55. 10.2340/16501977-0623 21031292

[13] Vermeulen J Neyens JCL van Rossum E Spreeuwenberg MD de Witte LP. Predicting ADL disability in community-dwelling elderly people using physical frailty indicators: a systematic review. BMC Geriatr. (2011) 11:33. 10.1186/1471-2318-11-33 21722355 PMC3142492

[14] Ludewig PM Borstad JD. Effects of a home exercise programme on shoulder pain and functional status in construction workers. Occup Environ Med. (2003) 60:841–9. 10.1136/oem.60.11.841 14573714 PMC1740414

[15] Khan F Pallant JF Brand C Kilpatrick TJ. Effectiveness of rehabilitation intervention in persons with multiple sclerosis: a randomised controlled trial. J Neurol Neurosurg Psychiatry. (2008) 79:1230–5. 10.1136/jnnp.2007.133777 18535027

[16] Briffa TG Eckermann SD Griffiths AD Harris PJ Heath MR Freedman SB Cost-effectiveness of rehabilitation after an acute coronary event: a randomised controlled trial. Med J Aust. (2005) 183(9):450–5. 10.5694/j.1326-5377.2005.tb07121.x 16274344

[17] Katajapuu N. Psychometric properties of 12-item world health organization disability assessment schedule 2.0 (WHODAS 2.0) amongst people with chronic musculoskeletal pain. Jyväskylä: JYU dissertations, University of Jyväskylä (2021).

[18] Ilves O. Rehabilitation after lumbar spine fusion—the effectiveness of a 12-month home exercise program. Jyväskylä: JYU dissertations, University of Jyväskylä (2020).

[19] Prince M Wimo A Guerchet M Ali G-C Wu Y-T Prina M. World alzheimer report 2015 the global impact of dementia. London: Alzheimer’s Disease International (2015). Available at: https://www.alzint.org/u/WorldAlzheimerReport2015.pdf (Accessed March 3, 2023).

[20] Jellinger K Danielczyk W Fischer Py. Clinicopathological analysis of dementia disorders in the elderly. J Neurol Sci. (1990) 95:239–58. 10.1016/0022-510X(90)90072-U 2358819

[21] Rios D Magasi S Novak C Hamiss M. Conducting accessible research: including people with disabilities in public health, epidemiological, and outcomes studies. Am J Public Health. (2016) 106:2137–44. 10.2105/AJPH.2016.303448 27736212 PMC5104996

[22] Baskerville R. What design science is not. Eur J Inf Syst. (2008) 17:441–3. 10.1057/ejis.2008.45

[23] van Aken J Romme G. Reinventing the future: adding design science to the repertoire of organization and management studies. Organ Manag J. (2009) 6:5–12. 10.1057/omj.2009.1

[24] ICF Research Branch. (2015). Available at: https://www.icf-research-branch.org/download/send/4-icf-core-sets/257-icf-rehabilitation-set (Accessed February 21, 2023).

[25] THL. (2022). Available at: https://thl.fi/en/web/functioning/toimia-functioning-measures-database (Accessed March 3, 2023).

[26] Valkeinen H Anttila H Kolehmainen L Lenkkeri K Mäkelä M Penttinen L (2020). Aikuisten toimintakyvyn itsearviointi kuntoutumistarpeen tunnistamisessa ja kuntoutumisen seurannassa. TOIMIA-suositus ID S026/1.4.2020.

[27] Lapin B Udeh B Bautista JF Katzan IL. Patient experience with patient-reported outcome measures in neurologic practice. Neurology. (2018) 91:e1135–51. 10.1212/WNL.0000000000006198 30135254

[28] Hansen ST Kjerholt M Christensen SF Brodersen J HølgeHazelton B. “I am sure that they use my PROM data for something important.” A qualitative study about patients’ experiences from a hematologic outpatient clinic. Cancer Nurs. (2020) 43(5):E273–82. 10.1097/NCC.0000000000000738 31361675

[29] Mahoney FI Barthel D. Functional evaluation: the barthel index. Md State Med J. (1965) 14:56–61. 14258950

[30] Jellinger K Danielczyk W Fischer P Gabriel E. Designing the national resident assessment instrument for nursing homes. Gerontologist. (1990) 30(3):293–307. 10.1093/geront/30.3.293 2354790

[31] Guralnik JM Simonsick EM Ferrucci L Glynn RJ Berkman LF Blazer DG A short physical performance battery assessing lower extremity function: association with self-reported disability and prediction of mortality and nursing home admission. J Gerontol. (1994) 49:M85–94. 10.1093/geronj/49.2.M85 8126356

[32] THL. (2021). Available at: https://thl.fi/en/web/thlfi-en/research-and-development/research-and-projects/national-finsote-survey (Accessed February 17, 2023).

[33] KELA. (2018). Available at: https://www.kela.fi/benefit-forms/GAS1.pdf

[34] Akpan A Roberts C Bandeen-Roche K Batty B Bausewein C Bell D Standard set of health outcome measures for older persons. BMC Geriatr. (2018) 18:36. 10.1186/s12877-017-0701-3 29394887 PMC5797357

[35] Kramer JM Schwartz A. Reducing barriers to patient-reported outcome measures for people with cognitive impairments. Arch Phys Med Rehabil. (2017) 98(8):1705–15. 10.1016/j.apmr.2017.03.011 28400180

[36] Chen A Väyrynen K Leskelä R-L Torkki P Heinonen S Tekay A The acceptability of implementing patient-reported measures in routine maternity care: a systematic review. Acta Obstet Gynecol Scand. (2023) 00:1–14. 10.1111/aogs.14506 PMC10008272 36647292

[37] Simpson JM Worsfold C Fisher KD Valentine JD. The CONFbal scale: a measure of balance confidence—a key outcome of rehabilitation. Physiotherapy. (2009) 95:103–9. 10.1016/j.physio.2008.12.004 19627691

[38] Yip AM Gorman MC Stadnyk K Mills WG MacPherson KM Rockwood K. A standardized menu for goal attainment scaling in the care of frail elders. Gerontologist. (1998) 38(6):735–42. 10.1093/geront/38.6.735 9868853

[39] Stolee P Rockwood K Fox RA Streiner DL. The use of goal attainment scaling in a geriatric care setting. J Am Geriatr Soc. (1992) 40(6):574–78. 10.1111/j.1532-5415.1992.tb02105.x 1587973

[40] Rockwood K Joyce B Stolee P. Use of goal attainment scaling in measuring clinically important change in cognitive rehabilitation patients. J Clin Epidemiol. (1997) 50(5):581–8. 10.1016/S0895-4356(97)00014-0 9180650

[41] Osoba D Rodrigues G Myles J Zee B Pater J. Interpreting the significance of changes in health-related quality-of-life scores. J Clin Oncol. (1998) 16:139–44. 10.1200/JCO.1998.16.1.139 9440735

[42] Malmivaara A. Real-effectiveness medicine—pursuing the best effectiveness in the ordinary care of patients. Ann Med. (2013) 2013(45):103–6. 10.3109/07853890.2011.653394 22380660

[43] Malmivaara A. Benchmarking controlled trial-a novel concept covering all observational effectiveness studies. Ann Med. (2015) 47:332–40. 10.3109/07853890.2015.1027255 25965700 PMC4673508

[44] Ravn MB Petersen KS Thuesen J. Rehabilitation for people living with dementia: a scoping review of processes and outcomes. J Aging Res. (2019) 2019. 10.1155/2019/4141050 31275651 PMC6589218

[45] Kudlicka A Martyr A Bahar-Fuchs A Sabates J Woods B Clare L. Cognitive rehabilitation for people with mild to moderate dementia. Cochrane Database Syst Rev. (2023) (6). 10.1002/14651858.CD013388.pub2 37389428 PMC10310315

